# The burden of very early dropout in infertility care: a nationwide population-based cohort study

**DOI:** 10.1093/humrep/dead226

**Published:** 2023-10-28

**Authors:** Khaoula Ben Messaoud, Jean Bouyer, Juliette Guibert, Elise de La Rochebrochard

**Affiliations:** Institut National d’Etudes Démographiques, Ined, Sexual and Reproductive Health and Rights Unit—UR14, Aubervilliers, France; Université Paris-Saclay, UVSQ, Inserm, CESP, Villejuif, France; Institut National d’Etudes Démographiques, Ined, Sexual and Reproductive Health and Rights Unit—UR14, Aubervilliers, France; Université Paris-Saclay, UVSQ, Inserm, CESP, Villejuif, France; Centre Médico-Chirurgical de la Baie de Morlaix, rond-point de la Vierge Noire, Morlaix, France; Institut National d’Etudes Démographiques, Ined, Sexual and Reproductive Health and Rights Unit—UR14, Aubervilliers, France; Université Paris-Saclay, UVSQ, Inserm, CESP, Villejuif, France

**Keywords:** infertility therapy, infertility epidemiology, infertility drugs, socioeconomic factors, access to health care, clomiphene, gonadotropins, patient dropouts, France

## Abstract

**STUDY QUESTION:**

What is the frequency and the associated factors of very early dropout following unsuccessful clomiphene citrate (CC)/gonadotropin treatment in the context of full coverage of treatment cost.

**SUMMARY ANSWER:**

Despite free treatment, almost one in four women had a very early dropout following unsuccessful CC/gonadotropin treatment, with patients below the poverty line being more likely to drop out early.

**WHAT IS KNOWN ALREADY:**

Success of infertility care is tarnished by very high dropout rates. Infertility care dropout has been considered as resulting principally from financial barriers because of the high cost of treatment. Nearly all previous work addressed dropout following IVF/ICSI. Factors associated with dropout following CC/gonadotropins may be different and also need to be investigated.

**STUDY DESIGN, SIZE, DURATION:**

Nationwide population-based cohort study.

**PARTICIPANTS/MATERIALS, SETTING, METHODS:**

Using the French national health insurance and hospital databases, we included in the cohort 27 416 women aged 18–49 years unsuccessfully treated with CC/gonadotropins in 2017. The main outcome was very early dropout, defined as discontinuation of all infertility treatment after unsuccessful treatment for 1–3 months. Very early treatment dropout was analysed by multivariate logistic regression.

**MAIN RESULTS AND THE ROLE OF CHANCE:**

Among women unsuccessfully treated with CC/gonadotropins, 22% dropped out of infertility care within 3 months. In multivariate analysis, higher early dropout following unsuccessful CC/gonadotropin treatment was associated with older and younger ages (≥35 and <25 years), being below the poverty line, being treated with CC prescribed by a general practitioner and lack of infertility tests or monitoring.

**LIMITATIONS, REASONS FOR CAUTION:**

This study is based on health administrative data that do not include reasons for dropout and record only a limited amount of information. It is thus not possible to analyse the reason for early dropout.

**WIDER IMPLICATIONS OF THE FINDINGS:**

Despite full coverage of all infertility treatment, women under the poverty line have a higher risk of very early dropout following unsuccessful CC/gonadotropin treatment. Better understanding is needed of the non-financial barriers and difficulties faced by these patients. To address disparities in infertility treatment, practitioner training could be reinforced to adapt to patients from different social and cultural backgrounds.

**STUDY FUNDING/COMPETING INTEREST(S):**

This work was supported by the ANR StimHo project, grant ANR-17-CE36-0011-01 from the French Agence Nationale de la Recherche. The authors have no conflict of interest to declare.

**TRIAL REGISTRATION NUMBER:**

N/A.

## Introduction

Nearly 50 million couples suffer from infertility worldwide, leading to a huge demand for infertility treatments (Boivin *et al.*, 2007; Mascarenhas *et al.*, 2012; Cox *et al.*, 2022). To treat infertility, ART have been increasingly used, with an estimate of nearly 3 million ART cycles conducted worldwide for the year 2014 (Chambers *et al.*, 2021). Infertility treatments also include ovulation induction using clomiphene citrate (CC) or gonadotropins (Carson and Kallen, 2021). Unlike ART, very little research has been carried out on CC/gonadotropin treatment, even though it is the first-line treatment for 8 in 10 infertile couples (Belgherbi and de La Rochebrochard, 2018; Carson and Kallen, 2021).

Although infertility treatments are increasingly used, their success is tarnished by very high dropout rates before achieving a pregnancy. In 34 studies published in 15 countries between 1995 and 2015, the overall dropout rate was estimated to be 50% during ART treatment, with large variations between countries from 21% to 64% (Arvis *et al.*, 2021). Dropouts occur as soon as after the first ART cycle with studies showing rates of 26% in France, 34% in the UK, 43% in Japan, and as high as 65% in the USA (Troude *et al.*, 2014; Smith *et al.*, 2015; Domar *et al.*, 2018; Hirakawa *et al.*, 2021). Dropouts probably also occur during CC/gonadotropin treatment but as yet there have been no published studies on this issue. Because of the high cost of infertility treatment, dropout has been considered as resulting principally from financial barriers (Domar *et al.*, 2018; Bedrick *et al.*, 2019; Ghorbani *et al.*, 2022). Moreover, some studies showed that when there are no or reduced financial barriers, infertile couples are more likely to access treatment and have a lower dropout rate (Vélez *et al.*, 2014; Crawford *et al.*, 2016; Bissonnette *et al.*, 2019). Nevertheless, even when infertility treatment costs are fully covered, high dropout rates have been observed during ART treatment (Troude *et al.*, 2014; Crawford *et al.*, 2016). Non-financial reasons for dropping out have been studied only for ART. They include psychological and physical burden, age of the woman, and personal conditions of life (Carson and Kallen, 2021; Ghorbani *et al.*, 2022). However, because CC/gonadotropin treatment is much less invasive than ART treatment, non-financial reasons for dropping out may be different than for ART. France offers a very interesting basis for exploring this issue, as costs of all infertility treatments, including CC/gonadotropins, IUI, IVF, and ICSI, are fully covered for the resident population by the national health insurance system.

Our aim was to explore very early dropout of all infertility treatment (including CC, gonadotropins, IUI, IVF, and ICSI) following unsuccessful CC/gonadotropin treatment in the French context of full treatment coverage in order to estimate its frequency and to analyse associated factors.

## Materials and methods

### Data source

In France, the national health insurance system covers 98% of the resident population. Its database exhaustively records all individual reimbursed drugs, medical devices, laboratory tests, medical procedures, alternative care, private and public hospital stays and care. It provides some information on the patient: sex, age, commune of residence and registration with the dedicated health insurance system for low-income patients (yes/no). This source has been described in detail elsewhere (Bezin *et al.*, 2017; Tuppin *et al.*, 2017). Access to French health insurance databases is strictly regulated by national legislation and is granted only by a personal time-limited accreditation for a specific research project.

### Study cohort

We included in the cohort all women (n = 27 416) aged 18–43 years who had a first infertility treatment with CC/gonadotropins in 2017 and who did not achieve pregnancy in the 2 years following the start of treatment.

### Outcome

The main outcome was very early dropout, defined as discontinuation of all infertility treatment (including CC, gonadotropins, IUI, IVF, and ICSI) after unsuccessful treatment for 1–3 months.

### Factors of early dropout

We studied the six following factors that were recorded in the French health insurance databases: the woman’s age, being below the poverty line, type of first-line fertility treatment (CC prescribed by a general practitioner (GP), CC prescribed by a gynaecologist, gonadotropins prescribed by a gynaecologist), infertility tests and monitoring (ultrasound examination, hormonal tests and cervical mucus testing).

The woman’s age was the age attained by the woman in 2017. Being below the poverty line (yes/no) was measured by registration with the dedicated health insurance system for low-income patients. This health insurance is granted to persons who have an annual household income below the poverty line (<50% of median annual income).

First-line infertility treatment was either CC or gonadotropins. CC may be prescribed by a gynaecologist or by a GP, whereas in France gonadotropins may only be prescribed by a gynaecologist. Prescriber and type of treatment could not therefore be included independently in analysis. A three-category variable was considered: CC prescribed by a GP, CC prescribed by a gynaecologist, gonadotropins prescribed by a gynaecologist.

Infertility tests and monitoring included three variables: ultrasound examination, hormonal tests, and cervical mucus testing.

### Statistical analysis

Very early treatment dropout was analysed in univariate and multivariate logistic regressions. All statistical analyses were performed using SAS Enterprise Guide software (version 4.3, SAS Institute Inc., Cary, NC, USA). The logistic procedure was used to estimate odds ratio (OR) and 95% CI.

## Results

Among the 27 416 women unsuccessfully treated with CC/gonadotropins, 22% had a very early dropout from all infertility treatments (including CC, gonadotropins, IUI, IVF, and ICSI) (Table 1).

**Table 1.** Description of the cohort study.

	Population	Percent
	(n = 27 416)	(%)
**Very early dropout**		
Yes	6129	22.4
No	21 287	77.6
**Age (years)**		
18–24	1848	6.7
25–29	6287	22.9
30–34	8190	29.9
35–39	6895	25.2
40–43	4196	15.3
**Below poverty line**		
Yes	3968	14.5
No	23 448	85.5
**Treatment type and prescriber**		
Clomiphene citrate prescribed by a gynaecologist	14 701	53.6
Gonadotropins prescribed by a gynaecologist	10 035	36.6
Clomiphene citrate prescribed by a GP	2680	9.8
**Ultrasound monitoring**		
Yes	18 676	68.1
No	8740	31.9
**Hormonal tests**		
Yes	18 913	69.0
No	8503	31.0
**Cervical mucus testing**		
Yes	1857	6.8
No	25 559	93.2

More than 50% of the study cohort was aged 30 to 39 years and almost 15% were under the poverty line (Table 1). The first treatment was CC prescribed by a gynaecologist for nearly 54% of women, gonadotropins prescribed by a gynaecologist for nearly 37%, and CC prescribed by a GP for less than 10%. In the study cohort, 32% of women had had ultrasound monitoring, 31% hormonal investigations, and 7% cervical mucus testing.

**Figure 1.**
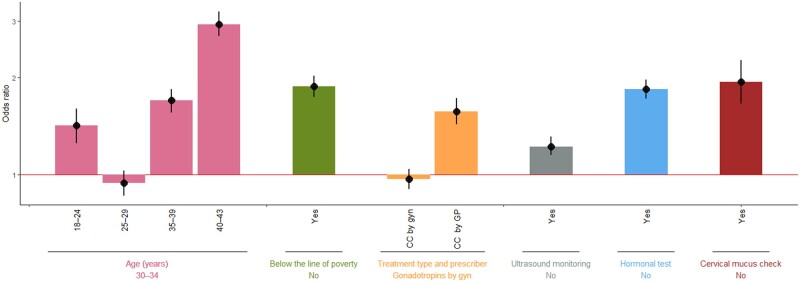
**Very early dropout from infertility treatment (n = 27** **416 French women unsuccessfully treated with clomiphene citrate/gonadotropins), summary of multivariate model.^a^** Odds ratios and 95% CI are shown in black, the reference category of each variable is presented horizontally below the name of the variable. CC: clomiphene citrate; GP: general practitioner; GYN: gynaecologist. ^a^Model presented in Table 2.

All six factors were significantly associated with very early dropout in both univariate and multivariate analysis (Fig. 1, Table 2). In multivariate analysis, very early dropout was significantly higher among the youngest and oldest women compared with women aged 30–34. Being below the poverty line was associated with very early dropout (OR 1.88; 95% CI 1.74; 2.03). There was no significant difference in very early dropout between women treated with CC versus gonadotropins prescribed by a gynaecologist, but very early dropout was significantly higher when the treatment was CC prescribed by a GP compared with gonadotropins prescribed by a gynaecologist (OR 1.57, 95% CI 1.43; 1.73). The three variables relating to infertility tests and monitoring showed the same trend, with a higher risk of very early dropout when there were no tests or monitoring.

**Table 2.** Univariate and multivariate analysis of very early dropout from infertility treatment (n = 27 416 French women unsuccessfully treated with Clomiphene citrate/gonadotropins).

Characteristics	Early dropout (%)	Univariate analysis			Multivariate analysis		
		Odds ratio	95% CI	*P*-value	Odds ratio	95% CI	*P*-value
**Age (years)**							
18–24	25.3	1.70	[1.51; 1.92]	<0.0001	1.42	[1.25; 1.60]	<0.0001
25–29	15.8	0.95	[0.87; 1.04]		0.94	[0.86; 1.03]	
30–34	16.5	1			1		
35–39	25.1	1.69	[1.56; 1.83]		1.70	[1.56; 1.84]	
40–43	37.6	3.04	[2.79; 3.31]		2.93	[2.69; 3.21]	
**Below poverty line**							
No	19.8	1			1		<0.0001
Yes	37.6	2.44	[2.27; 2.62]	<0.0001	1.88	[1.74; 2.03]	
**Treatment type and prescriber**							
Gonadotropins prescribed by gynaecologist	18.5	1			1		<0.0001
Clomiphene citrate prescribed by gynaecologist	21.6	0.82	[0.77; 0.88]	<0.0001	0.97	[0.90; 1.04]	
Clomiphene citrate prescribed by general practitioner	40.6	2.47	[2.26; 2.69]		1.57	[1.43; 1.73]	
**Ultrasound monitoring**							
Yes	27.1	1		<0.0001	1		<0.0001
No	20.2	1.47	[1.39; 1.56]		1.22	[1.15; 1.31]	
**Hormonal tests**							
Yes	17.5			<0.0001	1.		<0.0001
No	33.1	2.32	[2.19; 2.46]		1.84	[1.72; 1.97]	
**Cervical mucus testing**							
Yes	10.3	1		<0.0001	1		<0.0001
No	23.2	2.64	[2.27; 3.07]		1.94	[1.66; 2.27]	

## Discussion

In a context of free treatment of infertility care, almost one in four women (22%) had a very early dropout of all infertility treatment (including CC, gonadotropins, IUI, IVF, and ICSI) following unsuccessful CC/gonadotropin treatment. Higher early dropout was associated with older and younger ages, being below the poverty line, being treated with CC prescribed by a GP and lack of infertility testing or monitoring.

To the best of our knowledge, this is the first study estimating in a very large national sample very early dropout following unsuccessful CC/gonadotropin treatment. Only one Dutch study explored very early dropout among patients treated in one fertility centre at Jeroen Bosch Hospital: of the 440 women unsuccessfully treated with CC/gonadotropins/IUI, 15% discontinued care without using IVF/ICSI (Brandes *et al.*, 2009).

Our finding, highlighting a higher early dropout rate at older ages, is in line with a recent systematic review on reasons and factors associated with infertility treatment dropout (Ghorbani *et al.*, 2022). However, most studies explored dropout from IVF/ICSI treatment (Soullier *et al.*, 2011; Troude *et al.*, 2014; Dodge *et al.*, 2017; Domar *et al.*, 2018). Only one Dutch study based on pharmacy data from two large regions explored the use of CC treatment (Wang *et al.*, 2011). It observed a decreased number of treatment cycles of CC with increasing age of the woman, suggesting a possible association between early dropout and women’s age. For older women, early dropout may reflect awareness of the strong decrease in treatment success rate with increasing age (Soullier *et al.*, 2011; American College of Obstetricians and Gynecologists Committee on Gynecologic Practice and Practice Committee, 2014), leading to the patient and the doctor making a joint decision to stop infertility treatment (Soullier *et al.*, 2011; Seshadri *et al.*, 2021).

Our study also revealed for the first time an association between poverty and very early dropout following unsuccessful CC/gonadotropin treatment in a context of national full cost coverage of all infertility treatment. Poverty disproportionally affects immigrants and children of immigrants from Africa (39% and 27%) and from Asia (36% and 31%) compared to non-immigrant people with non-immigrant parents (11%) in France (Insee, 2023). The findings of this study likely reveal not only the effect of poverty but may also indicate the effect of other social disadvantages, such as immigration status and belonging to a racial or ethnic minority group. Ethnicity/race is an important component of social disadvantage that cannot be overlooked. Unfortunately, this information is not available for the French population due to the stringent data protection regulations in France. Two previous studies in the USA explored social disadvantage trough ethnicity/race, one in an Illinois fertility centre (Missmer *et al.*, 2011; Galic *et al.*, 2021a) and the second in a Massachusetts centre (Jain and Hornstein, 2005). They showed that even when treatment cost is fully covered, individuals belonging to a racial or ethnic minority group make less use of fertility care. The authors concluded that beyond treatment cost coverage, belonging to a minority group (the main poverty indicator in the USA) plays a key role in access to infertility care (Galic *et al.*, 2021a). More marginalized groups may have greater difficulty in facing the complexity of infertility care (Nachtigall *et al.*, 2009; Hoffman *et al.*, 2020; Cebert-Gaitors *et al.*, 2022). Some ethnic subgroups may also face language barriers (Nachtigall *et al.*, 2009; Galic *et al.*, 2021a), stigma (Vu *et al.*, 2022), and difficulties in managing bureaucracy related to infertility care (Missmer *et al.*, 2011). Such social mechanisms may partially underlie the association observed in our study between poverty and early dropout and would need further investigation.

Our study also showed an association between very early dropout and the type of care received: very early dropout was more frequent when the first treatment was CC prescribed by a GP and when the woman had no infertility tests or monitoring. This finding requires further research as it may have very important public health policy implications for infertility care. More specifically, it would be necessary to investigate how much GPs are aware of good practice for infertility treatment, as well as the reasons for omission of infertility tests and monitoring.

The main strength of this study is its analysis of a very large cohort drawn from the nationally exhaustive database of the French health insurance system. This is a major strength compared with most previous studies, which were usually based on ART patients from a single infertility centre (Domar *et al.*, 2018; Bedrick *et al.*, 2019). Another strength is that French health administrative data allow us to observe all infertility treatments followed by the woman everywhere in the country, whereas previous studies based on a single infertility centre usually classified as dropouts those patients who continued treatment in another fertility centre (Domar *et al.*, 2018). A limitation of our study is that health administrative data do not include reasons for dropout and record only a limited amount of information, far less than medical records from fertility centres. Another limitation is the small amount of information on the patients themselves, with no data on ethnicity for example. Thus, in the French health administrative data, the impact of the variable poverty may partially reflect the effect of other social disadvantage such as immigration status, and belonging from racial/ethnic minority group. Further study is needed to better understand the specific role of the different dimensions of social disadvantage in early dropout of infertility treatment.

### Implications and conclusion

In a national context of full coverage of all infertility treatments, the high rate of very early dropout following unsuccessful CC/gonadotropin treatment among women below the poverty line highlights the need to better understand non-financial barriers and difficulties faced by these patients. It also raises questions on the need for better training for practitioners to enable them to adapt to patients from different social and cultural backgrounds (Missmer *et al.*, 2011; Galic *et al.*, 2021b), provide more patient-centred care and clearer information, and improve partnership building and patient engagement in communication processes (Shen *et al.*, 2018; Gameiro *et al.*, 2019). Education and outreach should be considered as essential to address disparities in infertility care (Insogna *et al.*, 2020).

## Data Availability

To request access to French health insurance database, please contact the Health Data Hub (website: https://www.health-data-hub.fr/) in accordance with French law (National Decree no. 2016-316, 13 October 2016). The SAS code underlying this article will be shared on reasonable request to the corresponding author.
